# *EPCR* Gene Ser219Gly Polymorphism and Venous Thromboembolism: A Meta-Analysis of 9,494 Subjects

**DOI:** 10.3389/fphys.2017.00339

**Published:** 2017-05-26

**Authors:** Yan-yan Li, Jing-jing Wu, Xin-xing Yang, Hong-yu Geng, Ge Gong, Hyun Jun Kim

**Affiliations:** ^1^Department of Gerontology, First Affiliated Hospital of Nanjing Medical UniversityNanjing, China; ^2^Department of Nephrology, First Affiliated Hospital of Nanjing Medical UniversityNanjing, China; ^3^Department of Gerontology, Nanjing General HospitalNanjing, China; ^4^Department of Physiology, University of CincinnatiCincinnati, OH, United States

**Keywords:** *EPCR*, Ser219Gly, polymorphism, venous thromboembolism, genetic

## Abstract

**Background:** Although *endothelial cell protein C receptor (EPCR)* gene Ser219Gly polymorphism has been associated with venous thromboembolism (VTE) susceptibility, no clear consensus has yet been reached.

**Objective and methods:** A meta-analysis of 9,494 subjects from 13 individual studies was conducted to better elucidate the potential relationship between the *EPCR* gene Ser219Gly polymorphism and VTE. Pooled odds ratios (ORs) and their corresponding 95% confidence intervals (CIs) were evaluated by using fixed or random effect models.

**Results:** The current meta-analysis suggested that there was a significant association between *EPCR* gene Ser219Gly polymorphism and VTE under allelic (OR: 1.42, 95% CI: 1.21–1.66, *P* = 1.30 × 10^−5^), recessive (OR: 2.02, 95% CI: 1.44–2.85, *P* = 5.35 × 10^−5^), homozygous (OR: 2.24, 95% CI: 1.59–3.16, *P* = 3.66 × 10^−6^), and additive genetic models (OR: 1.63, 95% CI: 1.30–2.04, *P* = 2.24 × 10^−5^).

**Conclusions:** *EPCR* gene Ser219Gly polymorphism was associated with an elevated risk of VTE and the Gly residue carriers of the *EPCR* gene might be predisposed to VTE.

## Introduction

Venous thromboembolism (VTE) encompasses deep vein thrombosis (DVT) and pulmonary empolism (PE). DVT indicates the formation of a venous thrombus typically in the deep veins of the legs, which may undergo embolization when the clot is dislodged from the underlying endothelium and carried downstream by circulatory system potentially reaching the lungs where it can cause a sudden occlusion of pulmonary circulation, referred to as PE. This event presents as a clinical emergency that can endanger the patient's life. In Western countries, VTE morbidity is around 1–2% in the whole population and rising. The early mortality of the DVT and PE are as high as 3.8 and 38.9%, respectively (Naess et al., 2007).

Hypercoagulability plays a major role in clot formation and can be mediated by impaired functioning of anti-coagulant pathways, such as the Protein C system. Protein C interacts with the endothelial protein C receptor (EPCR) expressed on the endothelium of the great vessels to cleave plasminogen to plasmin, a potent anti-coagulant (Castellino and Ploplis, 2009). EPCR is a Vitamin K-dependent Type-1 transmembrane protein that exhibits the same homology as the MHC class I/CD1 family of proteins (Oganesyan et al., 2002). It can also exist in a soluble form in the plasma (sEPCR) when cleaved by matrix metalloproteinases (MMP) (Xu et al., 2000; Bae et al., 2007).

Gene mutations in the *EPCR* gene, which is located on 20q11.2, may push hemostasis toward hypercoagulable state. It spans 6 kb, encodes for 241 amino acids, and is organized into 4 exons and 3 introns (Li et al., 2015). The Ser219Gly polymorphism that we are interested in involves an A-G base transition at either the 6,936th or 4,600th position. This mutation results in a Gly-Gly structure in the transmembrane region of the protein that could potentially reduce the stability of the helical structure and make it more vulnerable to MMP-mediated cleavage. Decreasing the amount of EPCR expressed on the cytomembrane could limit Protein C activity, resulting in a pro-thrombotic state in the body (Chen et al., 2011).

The frequent of Ser219Gly polymorphism in the general population is about 12% (Wang and Hu, 2008). Many studies have been conducted to elucidate the relationship between the *EPCR* Ser219Gly gene polymorphism and VTE, but the research has yet to present a clear consensus. In 2015, Sun et al. found that individuals with DVT exhibited higher Gly residue frequencies and concluded that the *EPCR* gene Ser219Gly polymorphism was associated with the DVT risk in a Chinese population (Sun and Yin, 2015). Looking at an Egyptian population, Zoheir et al. also found that mutant genotypes of *EPCR* gene 6936AG polymorphism (AG, GG) were associated with an increased risk for DVT as well as its mutant allele G (Zoheir et al., 2016). On the other hand, Anastasiou et al. found prevalence of the rare *EPCR* 219Gly residues to be comparable between patients with thrombotic disorders and controls in a Greek population. The *EPCR* gene Ser219Gly polymorphism seemed to have no impact on VTE recurrence (Anastasiou et al., 2016).

To determine whether the *EPCR* gene Ser219Gly polymorphism was associated with VTE susceptibility, the current meta-analysis of 9,494 subjects from 13 individual studies was conducted (Supplements S1).

## Materials and methods

### Publication search and inclusion criteria

We conducted a primary search using the terms “*EPCR*,” “Ser219Gly,” “6936A/G,” “4600A/G,” “rs867186,” “venous thromboembolism,” and “polymorphism,” through PubMed, WanFang database, the VIP database, the China National Knowledge Infrastructure, Embase, and the Web of Science. This search yielded papers published between 2004 and 2016 with the most recent update occurring on May 06, 2017.

The following inclusion criteria had to be met by the selected studies for our meta-analysis. Studies selected must: (a) assess the association of VTE and *EPCR* gene Ser219Gly polymorphism, (b) diagnose DVT by Doppler ultrasonography of the lower extremities, or diagnose PE by compression or ventilation lung ultrasonography, contrast venography, lung ventilation/perfusion scan, conventional pulmonary angiography, or computed tomographic angiography, (c) have control groups at Hardy-Weinberg equilibrium (HWE). The studies must be officially published cohort or case-control studies.

### Data extraction

The data was abstracted according to a standardized protocol by three authors. Two were responsible for removing duplicate studies while the third acted as an arbitrator to resolve any disagreements. Studies that did not meet inclusion criteria, were published repeatedly, or did not supply sufficient data were removed. Similar data sets presented in multiple publications by a single author group were represented once in the current meta-analysis. The listed data table also had to include items such as the first author's name, publication year, region, genotyping method, matching criteria, the genotype number in the VTE and control groups, and sample size of VTE and controls (Table 1).

**Table 1:** **Characteristics of the investigated studies of the association between the ***endothelial cell protein C receptor (EPCR)*** gene rs867186 polymorphism and venous thromboembolism (VTE)**.

**Author**	**Year**	**Region**	**Ethnicity**	**VTE**			**Control**			**Matching criteria**	**sample size (VTE/control)**
				**AA**	**AG**	**GG**	**AA**	**AG**	**GG**		
Yin GC (Yin et al., 2012)	2012	China	Chinese	69	38	3	89	22	1	Age, sex, ethnicity	110/112
Chen XD (Chen et al., 2011)	2011	China	Chinese	49	15	1	63	7	1	Age, sex, ethnicity	65/71
Sun XL (Sun and Yin, 2015)	2015	China	Chinese	43	28	4	52	12	1	Age, sex, ethnicity	75/65
Wang XB (Wang and Hu, 2008)	2008	China	Chinese	71	37	2	84	26	0	Age, sex, ethnicity	110/110
Zoheir N (Zoheir et al., 2016)	2016	Egypt	Non-Chinese	24	58	8	54	34	2	Age, sex, ethnicity	90/90
Saposnik B (Saposnik et al., 2004)	2004	France	Non-Chinese	249	85	4	278	58	2	Age, sex, ethnicity	338/338
Uitte dW (Uitte de Willige et al., 2004)	2004	Netherlands	Non-Chinese	345	116	10	361	100	10	Age, sex, ethnicity	471/471
Medina P (Medina et al., 2005)	2005	Spain	Non-Chinese	77	17	1	145	35	1	Age, sex, ethnicity	95/181
Pecheniuk NM (Pecheniuk et al., 2008)	2008	USA	Non-Chinese	82	27	5	87	24	3	Age, sex, ethnicity	114/114
Trégouët DA (Trégouët et al., 2009)	2009	France	Non-Chinese	1194	314	25	1657	357	13	Age, sex, ethnicity	1533/2027
Heit (Dennis et al., 2012)	2012	USA	Non-Chinese	978	264	28	1029	257	16	Age, sex, ethnicity	1270/1302
Karabıyık A (Karabıyık et al., 2012)	2012	Turkey	Non-Chinese	75	33	3	51	21	1	Age, sex, ethnicity	111/73
Anastasiou G (Anastasiou et al., 2016)	2016	Greece	Non-Chinese	50	8	0	82	18	0	Age, sex, ethnicity	58/100

*EPCR, endothelial cell protein C receptor; PCR-RFLP, Polymerase chain reaction-restriction fragment length polymorphism; PCR-RFLP method and Case-control study design were adopted in the above studies*.

### Statistical analyses

Four genetic models were used in the current meta-analysis: allelic (G allele distribution frequency), recessive (GG vs. AG and AA), homozygous (GG vs. AA), and additive (G vs. A). The relationship of *EPCR* gene Ser219Gly polymorphism and VTE was compared by using odds ratios (ORs) and their corresponding 95% confidence intervals (CIs). Chi-square-based Q-tests were used to calculate the heterogeneity among the studies with significance set at *P* < 0.05 level (Cochran, 1968). If no heterogeneity was detected among the included studies, the fixed-effect model (the Mantel–Haenszel method) would be used (Mantel and Haenszel, 1959). Otherwise, the random-effect model (the DerSimonian and Laird method) would be used (DerSimonian and Laird, 1986). *Z*-test was used to assess the pooled OR and the significance was set at *P* < 0.05 level. The effects of population stratification have also been conducted. The sensitivity analysis has been performed to determine the pooled results stability.

The Fisher's exact test was used to evaluate the HWE and the significance was set at *P* < 0.05 level. The funnel plot was used to assess the potential publication bias. The Egger's linear regression test on a natural log scale of the OR was used to evaluate the funnel plot symmetry and the significance was set at *P* < 0.05 level (Egger et al., 1997). The softwares Stata 12.0 and Review Manager 5.0 were used to perform the statistical analyses (StataCorp, College Station, TX, USA).

## Results

### Studies and populations

**Figure 1:**
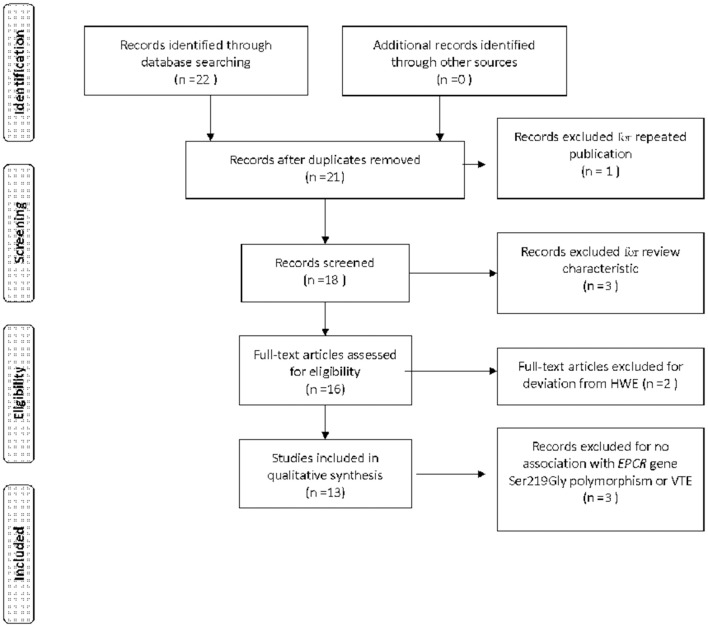
**PRISMA 2009 Flow Diagram describing the included/excluded literatures**.

Data was extracted from 4,440 VTE cases and 5,054 controls (Table 1) (Saposnik et al., 2004; Uitte de Willige et al., 2004; Medina et al., 2005; Pecheniuk et al., 2008; Wang and Hu, 2008; Trégouët et al., 2009; Chen et al., 2011; Dennis et al., 2012; Karabıyık et al., 2012; Yin et al., 2012; Sun and Yin, 2015; Anastasiou et al., 2016; Zoheir et al., 2016). Of the 22 papers acquired through the initial retrieval process, 13 were recruited for the current meta-analysis. Among the nine rejected studies, three papers were reviews and two did not meet HWE (Medina et al., 2004; Navarro et al., 2008). One paper was published by the same author group to that by Medina et al. in 2005 (Yamagishi et al., 2009). Three papers were not related to the topic at hand (Figure 1).

### Pooled analyses

**Figure 2:**
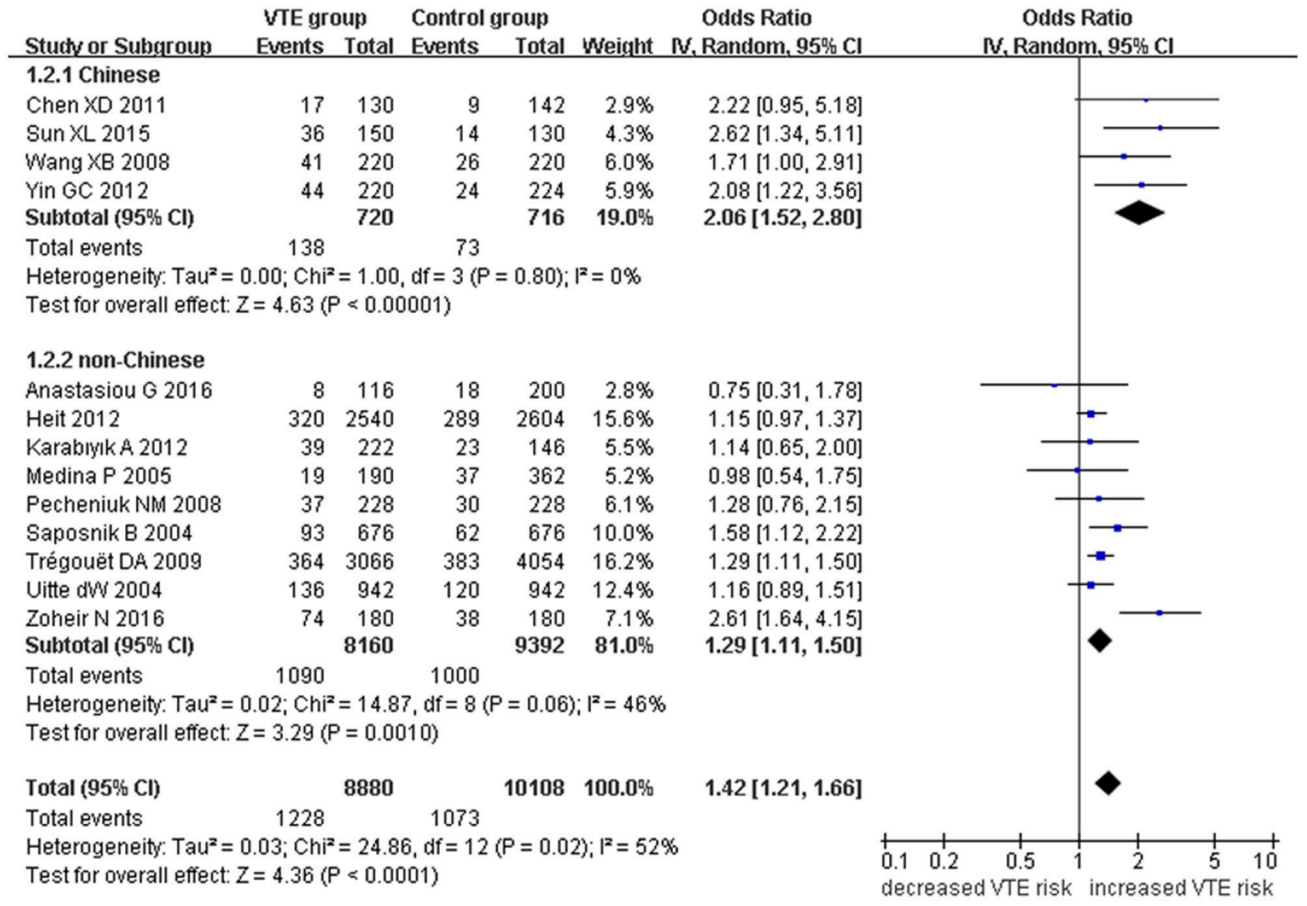
**Forest plot of VTE associated with ***EPCR*** gene Ser219Gly polymorphism under an allelic genetic model (distribution of Gly residue frequency of ***EPCR*** gene Ser219Gly polymorphism)**.

**Figure 3:**
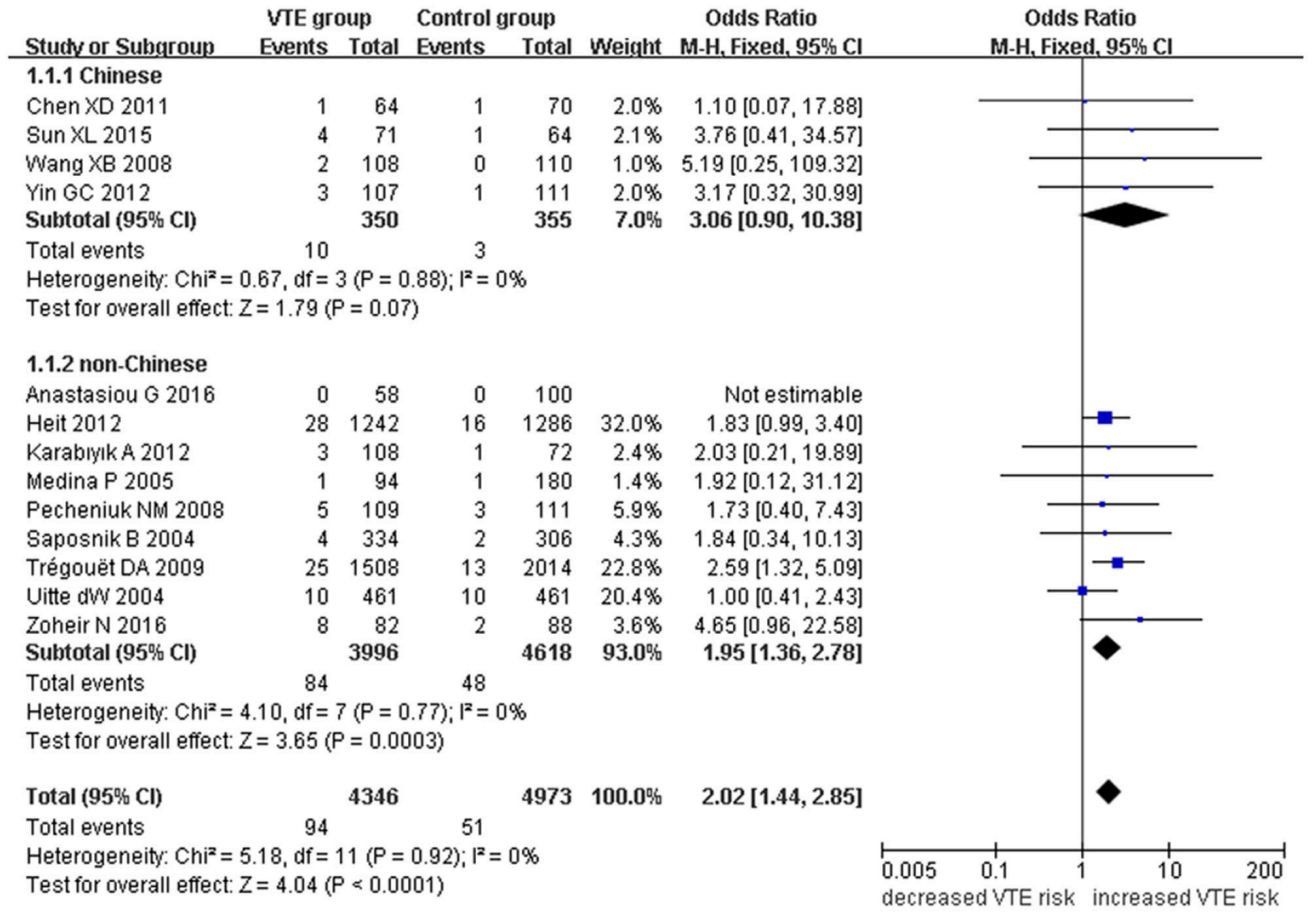
**Forest plot of VTE associated with ***EPCR*** gene Ser219Gly polymorphism under a recessive genetic model (GG vs. AA + AG)**.

**Figure 4:**
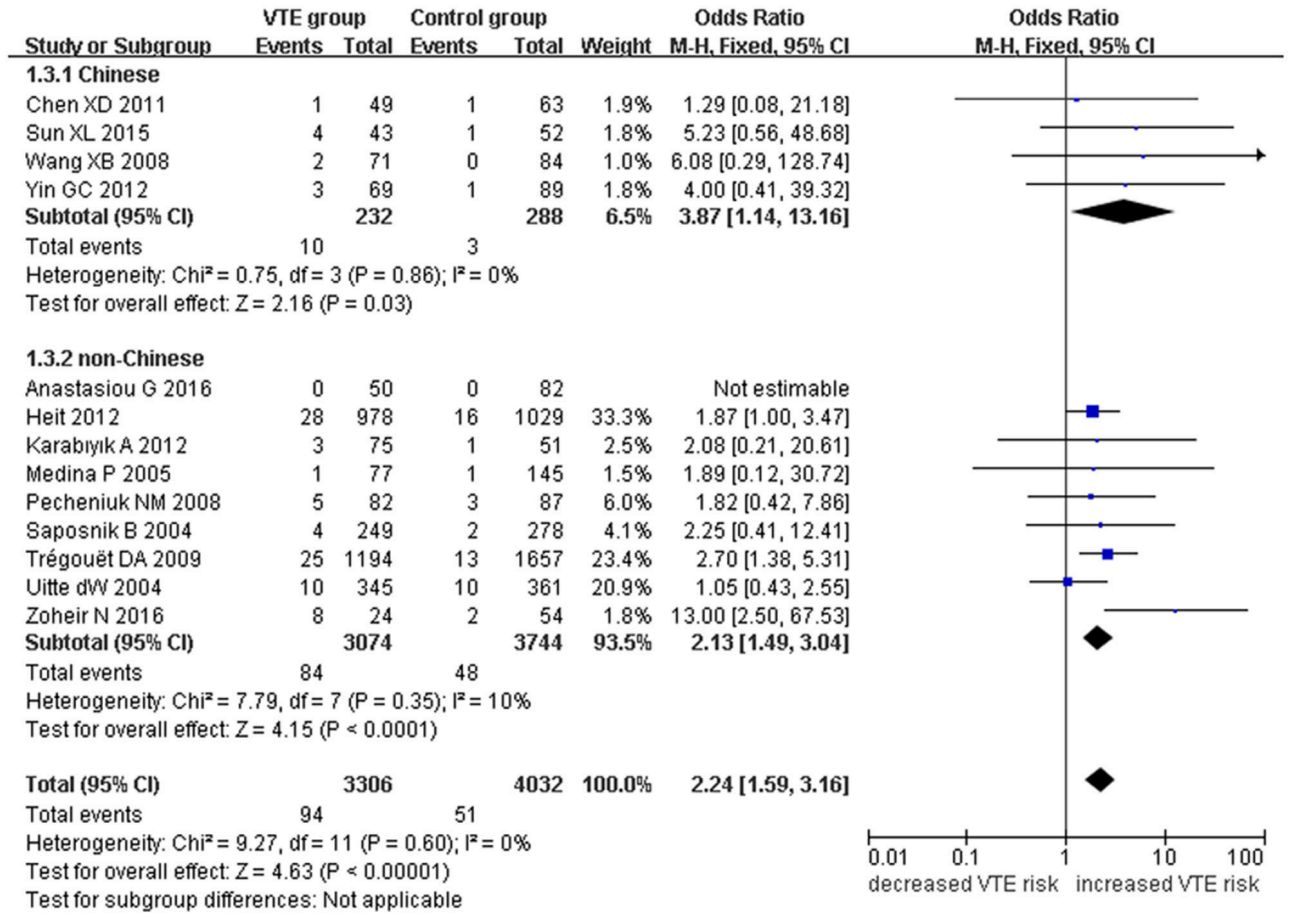
**Forest plot of VTE associated with ***EPCR*** gene Ser219Gly polymorphism under a homozygous genetic model (GG vs. AA)**.

**Figure 5:**
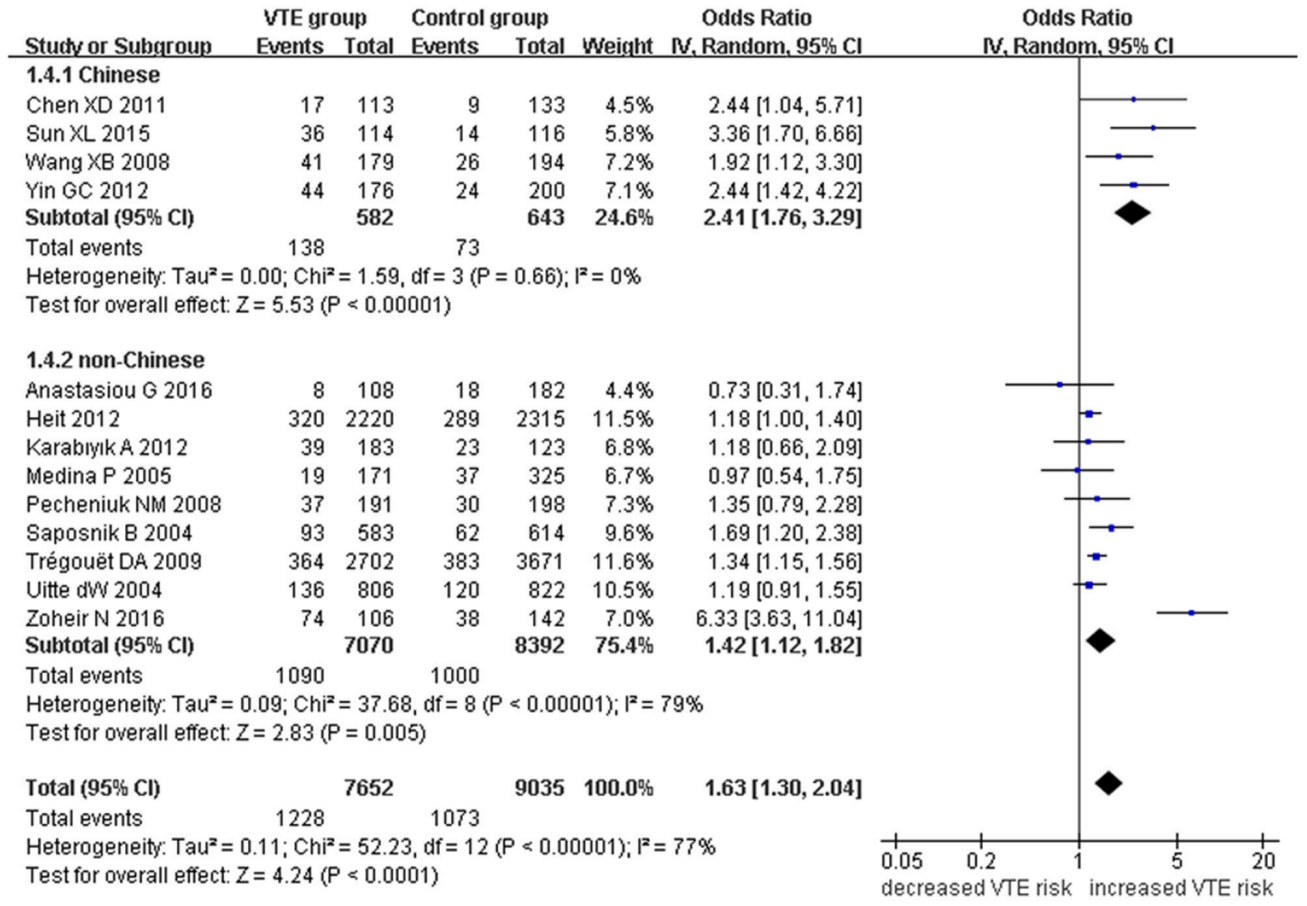
**Forest plot of VTE associated with ***EPCR*** gene Ser219Gly polymorphism under an additive genetic model (total G allele vs. total A)**.

Our meta-analysis suggests a significant association between the *EPCR* gene Ser219Gly polymorphism and VTE under allelic (OR: 1.42, 95% CI: 1.21–1.66, *P* = 1.30 × 10^−5^), recessive (OR: 2.02, 95% CI: 1.44–2.85, *P* = 5.35 × 10^−5^), homozygous (OR: 2.24, 95% CI: 1.59–3.16, *P* = 3.66 × 10^−6^), and additive genetic models (OR: 1.63, 95% CI: 1.30–2.04, *P* = 2.24 × 10^−5^, Table 2, Figures 2–5).

**Table 2:** **Summary of meta-analysis of association between ***endothelial cell protein C receptor (EPCR)*** gene rs867186 polymorphism and VTE**.

**Genetic model**	**Pooled OR (95% CI)**	**Z value**	**P value**	**Literature number**	**VTE size**	**control size**	***P*_heterogeneity(*I*^2^%)_**
Allelic genetic model	1.42 (1.21–1.66)	4.36	1.30 × 10^−5^*^^	13	4,440	5,054	0.02^*^(52%)
Chinese subgroup	2.06 (1.52–2.80)	4.63	3.66 × 10^−6^*^^	4	360	358	0.80 (0%)
Non-Chinese subgroup	1.29 (1.11–1.50)	3.29	0.001^*^	9	4,080	4,696	0.06 (46%)
Recessive genetic model	2.02 (1.44–2.85)	4.04	5.35 × 10^−5^*^^	13	4,440	5,054	0.92 (0%)
Chinese subgroup	3.06 (0.90–10.38)	1.79	0.07	4	360	358	0.88 (0%)
Non-Chinese subgroup	1.95 (1.36–2.78)	3.65	0.0003^*^	9	4,080	4,696	0.77 (0%)
Homo genetic model	2.24 (1.59–3.16)	4.63	3.66 × 10^−6^*^^	13	4,440	5,054	0.60 (0%)
Chinese subgroup	3.87 (1.14–13.16)	2.16	0.03^*^	4	360	358	0.86 (0%)
Non-Chinese subgroup	2.13 (1.49–3.04)	4.15	3.32 × 10^−5^*^^	9	4,080	4,696	0.35 (10%)
Additive genetic model	1.63 (1.30–2.04)	4.24	2.24 × 10^−5^*^^	13	4,440	5,054	<0.00001^*^ (77%)
Chinese subgroup	2.41 (1.79–3.29)	5.53	3.20 × 10^−8^*^^	4	360	358	0.66(0%)
Non-Chinese subgroup	1.42 (1.12–1.82)	2.83	0.005^*^	9	4,080	4,696	<0.00001^*^ (79%)

* *P ≤ 0.05. EPCR, endothelial cell protein C receptor; VTE, thromboembolism; CI, confidence interval; OR, odds ratio; VTE size, the total number of VTE cases; control size, the total number of control group; Allelic genetic model, G allele distribution frequency; Recessive genetic model, GG vs. AA + AG; Homozygous genetic mode, GG vs. AA; Additive genetic model, total G allele vs. total A*.

We also analyzed subgroups, stratified by ethnicity. In the Chinese subgroup, we found significant association between *EPCR* gene Ser219Gly polymorphism and VTE under allelic (OR: 2.06, 95% CI: 1.52–2.80, *P* = 3.66 × 10^−6^), homozygous (OR: 3.87, 95% CI: 1.14–13.16, *P* = 0.03), and additive genetic models (OR: 2.41, 95% CI: 1.76–3.29, *P* = 3.20 × 10^−8^). No significant association was detected under the recessive genetic model (OR: 3.06, 95% CI: 0.90–10.38, *P* = 0.07).

In the non-Chinese subgroup, a significant association between them was also observed under allelic (OR: 1.29, 95% CI: 1.11–1.50, *P* = 0.001), recessive (OR: 1.95, 95% CI: 1.36–2.78, *P* = 0.0003), homozygous (OR: 2.13, 95% CI: 1.49–3.04, *P* = 0.03), and additive genetic models (OR: 1.42, 95% CI: 1.12–1.82, *P* = 0.005).

In the whole population, no significant heterogeneity was detected under recessive or homozygous genetic models (*P* > 0.05). However, allelic and additive genetic models exhibited significant heterogeneity (*P* > 0.05). In the subgroup analysis, no significant heterogeneity was detected under all of the genetic models in the Chinese population (*P* > 0.05). Regard to the non-Chinese population, heterogeneity was detected under the additive genetic models (*P* < 0.05). This suggests that the source of heterogeneity was associated with ethnicity.

### Sensitivity analysis

**Figure 6:**
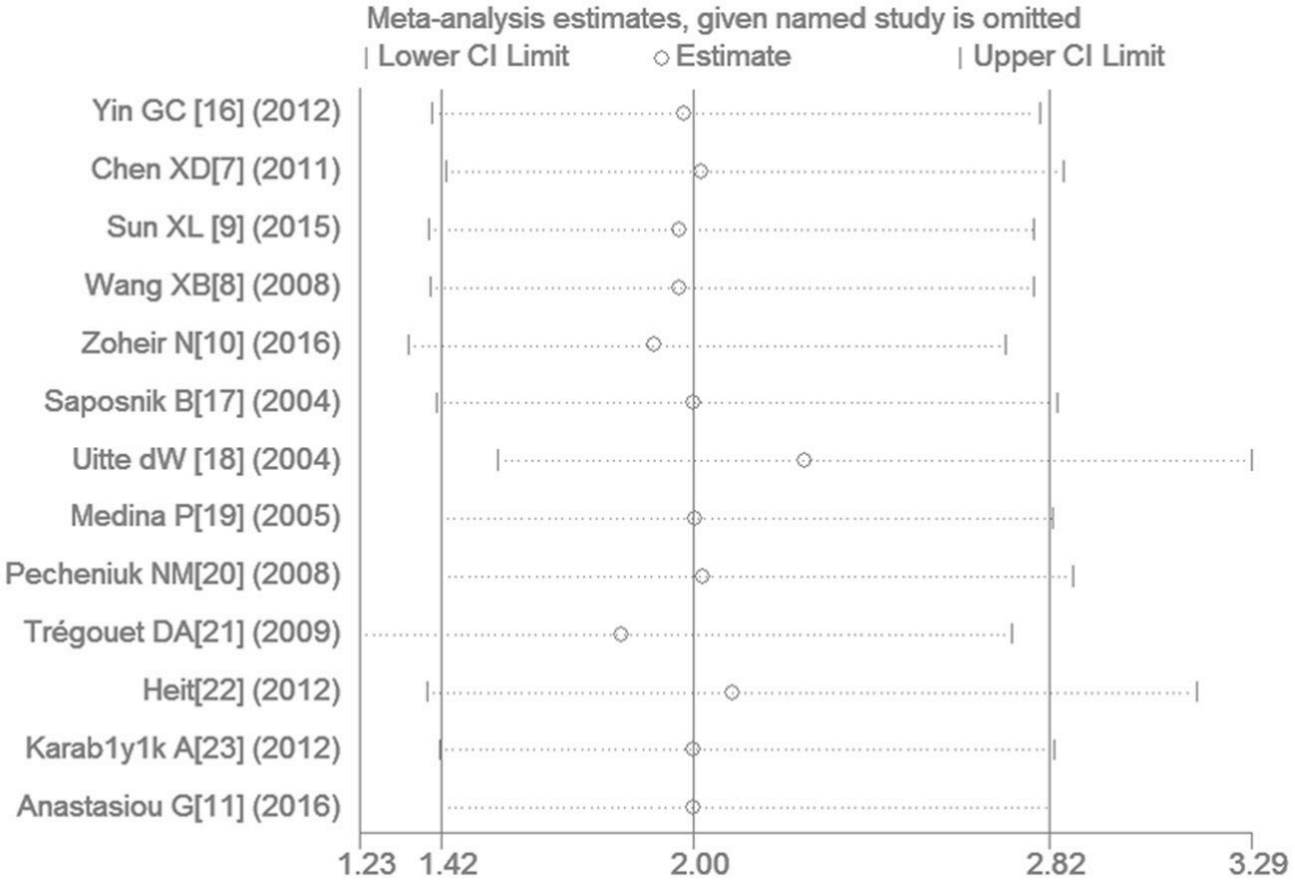
**The sensitivity analysis for studies of the association of VTE and ***EPCR*** gene Ser219Gly polymorphism under recessive genetic model (GG vs. AA + AG)**.

Removing the study by Zoheir et al. (2016) eliminated the heterogeneity detected in the non-Chinese population while still providing results consistent with our initial findings. The sensitivity analysis has shown that a more significant association between them was found after the study by Uitte de Willige et al. (2004) was omitted under the recessive genetic model (OR: 2.26, 95% CI: 1.55–3.29). However, removing any study in the current meta-analysis does not generate the inconsistent results with the initial findings (Figure 6).

### Bias diagnostics

**Figure 7:**
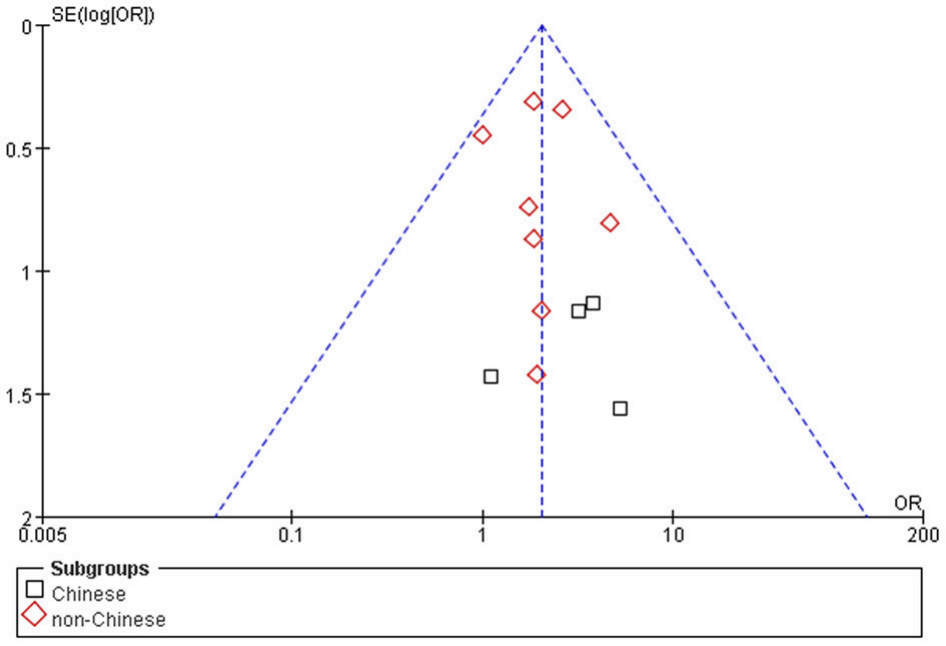
**The funnel plot for studies of the association of VTE and ***EPCR*** gene Ser219Gly polymorphism under recessive genetic model (GG vs. AA + AG)**. The horizontal and vertical axis correspond to the OR and confidence limits. OR, odds ratio; SE, standard error.

**Figure 8:**
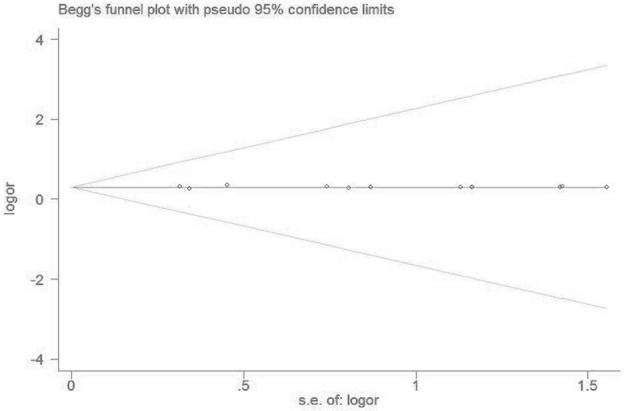
**The Begg's funnel plot for studies of the association of VTE and ***EPCR*** gene Ser219Gly polymorphism under recessive genetic model (GG vs. AA + AG)**. The horizontal and vertical axis correspond to the LogOR and confidence limits. OR, odds ratio; SE, standard error.

No visible publication bias has been detected in the funnel plot under the recessive genetic model (Figure 7). Furthermore, no significant difference was found in the Egger's test, which implied that there was no publication bias in this meta-analysis under recessive genetic model (*T* = 1.13, *P* = 0.283, Figure 8).

## Discussion

In the current meta-analysis, we found a significant association between the *EPCR* gene Ser219Gly polymorphism and VTE under allelic (OR: 1.42), recessive (OR: 2.02), homozygous (OR: 2.24), and additive genetic models (OR: 1.63). This suggests that the *EPCR* gene Ser219Gly polymorphism is associated with an elevated risk of VTE and that carriers of the G allele of the *EPCR* gene at the 6,936th or 4,600th position may be predisposed to VTE. Our analysis of the data by ethnicity echoed our initial findings with significant association detected in both the non-Chinese and Chinese ethnicities. Although heterogeneity was detected under allelic, and additive genetic models in the whole population (P_heterogeneity_ < 0.05), our subgroup analysis found it to be limited to the non-Chinese group, suggesting ethnicity to be the main source of heterogeneity (P_heterogeneity_ > 0.05).

EPCR is primarily expressed on the endothelial apical cell surface of the big vessels, but is also found in relatively high amounts in the placenta, lung, liver, and heart (Chen and Yuan, 2007; Iverson and Gomez, 2013). It also plays a role in many biological processes. Its canonical role is in the amplification of thrombin-activated Protein C activity, but EPCR has also demonstrated anti-apoptotic and inflammatory properties through the inhibition of the protease-activated receptor 1 and neutrophil granulocyte adhesion molecule CD11b/CD18, respectively (Riewald et al., 2002; Joyce et al., 2004).

*In vitro* and *in vivo* experiments show that EPCR expression is inhibited by various molecules, such as lipopolysaccharide (LPS), interleukin-1â (IL-1â), TNF-α, and thrombin. EPCR is also subject to MMP-mediated cleavage with the resultant sEPCR contributing to a pro-coagulation state through two mechanisms. Firstly, it acts as a competitive inhibitor by sEPCR by maintaining its interactions with activated Protein C, but no longer contributing to the thrombin-thrombomodulin (TM) system (Yin and Jin, 2013). sEPCR can also induce conformational changes in the APC active sites that reduce its ability to inhibit coagulation factor Va (Liaw et al., 2000).

The role of *EPCR* gene Ser219Gly mutation in facilitating the removal of EPCR from the cytomembrane and increasing levels of sEPCR in the plasma was fully verified through *in vitro* experiments. In a cell line with the Ser219Gly polymorphism, the EPCR drop rate was 5–7 times that of a WT cell line when stimulated with myristic acid-phorbol-acetic acid ester (Qu et al., 2006). This increase was also correlated to a hypercoagulation state.

We believe that the current meta-analysis offers an improved perspective on the subject. In 2012, Dennis et al. have performed a meta-analysis on the relationship between *EPCR* gene Ser219Gly variant and common thrombotic disorders risk (Dennis et al., 2012) that found a significant association between the *EPCR* rs867186 variant and VTE, but their analysis included studies by Medina et al. in 2004 and Yamagishi et al. in 2009 that had non-HWE controls (Medina et al., 2004; Yamagishi et al., 2009). The individual study by Navarro et al. (2008) was performed by the same author group to the study by Medina et al. (2005). However, both were adopted by their meta-analysis. By excluding studies that had non-HWE populations and adopting studies conducted by the same author groups to a single application, we believe our meta-analysis presents a more dependable conclusion. Our study also had broad representation of the Chinese and non-Chinese population. All four studies performed in Chinese population were used in our meta-analysis (Egger et al., 1997; Wang and Hu, 2008; Chen et al., 2011; Sun and Yin, 2015) and all studies in the non-Chinese population published after 2012 were also included in the current meta-analysis (Karabıyık et al., 2012; Anastasiou et al., 2016; Zoheir et al., 2016).

This study is not without limitations, however. We still lack the multiple large-scale studies necessary to completely understand the relationship between the *EPCR* gene Ser219Gly polymorphism and VTE. Furthermore, environmental factors that play significant roles in VTE development, such as trauma and surgery, were not controlled for. The micro-effects of many other related genes have yet to be fully understood (i.e., *Methylenetetrahydropholate reductase* gene C677T polymorphism, the *coagulation factor XI* gene rs2289252 and rs2036914 loci polymorphism) (Yin and Jin, 2013; Zhang et al., 2016).

In conclusion, *EPCR* gene Ser219Gly polymorphism was significantly associated with VTE susceptibility. The persons with the Gly residue of *EPCR* gene Ser219Gly polymorphism might be susceptible to VTE. More studies on the relationship between them should be carried out to further clarify this conclusion.

## Author contributions

YL and JW researched data. YL and HK wrote manuscript, researched data. YL, HG, and XY reviewed/edited manuscript. YL contributed to discussion, reviewed/edited manuscript. YL and GG researched data, contributed discussion.

### Conflict of interest statement

The authors declare that the research was conducted in the absence of any commercial or financial relationships that could be construed as a potential conflict of interest.
